# Lower Grade Gliomas

**DOI:** 10.1007/s11910-020-01040-8

**Published:** 2020-05-22

**Authors:** Gilbert Youssef, Julie J. Miller

**Affiliations:** Pappas Center for Neuro-Oncology, Department of Neurology, Massachusetts General Hospital, Boston, MA USA

**Keywords:** Low-grade gliomas, Astrocytomas, Oligodendrogliomas, IDH mutation, 1p/19 codel, IDH inhibitors, PARP inhibitors

## Abstract

**Purpose of Review:**

Low-grade gliomas (LGG) are a group of primary brain tumors that arise from supporting glial cells. They are characterized by a mutation in the isocitrate dehydrogenase (IDH) enzyme and include astrocytomas and oligodendrogliomas. They usually affect young adults, and their main treatment consists of surgical resection, followed by radiation and chemotherapy in selected patients. This article reviews recent research on the clinical and molecular aspects of the disease and innovative therapeutic modalities in the process.

**Recent Findings:**

Newly identified clinical and molecular features are currently used in the classification of LGG and applied in treatment-planning decisions. Advanced studies on the cellular level have an advanced understanding of the metabolic effects induced by IDH mutations, offering opportunities for specific targeted therapies that may improve patient outcomes. Such findings may lead to a paradigm shift in the treatment of these tumors.

**Summary:**

Although LGG are sensitive to radiation and chemotherapy, these therapies are not curative, and patient survival remains limited, raising the need for more creative and effective interventions.

## Introduction

Low-grade gliomas (LGG) constitute a heterogeneous group of neuroepithelial neoplasms arising from the supporting glial cells of the central nervous system (CNS). Classically, gliomas have been classified by the World Health Organization (WHO) into four grades, depending on their histopathological features, and only WHO grade I and II gliomas were considered low-grade [1]. These two subcategories were not only different histologically, with WHO grade II characterized by the presence of atypia, but they also had different clinical patterns. Indeed, WHO grade I tumors are benign and usually occur in children, whereas WHO grade II gliomas are rarely curable and frequently transform into higher grade tumors [2]. However, in 2014, the International Society of Neuropathology established guidelines to implement molecular parameters in the classification of CNS tumors, and the newest WHO classification of CNS tumors, published in 2016, combined both histopathologic and genotypic features in the classification of these tumors [3, 4•]. Furthermore, in this classification, the molecular phenotype trumps the histopathological one and depends primarily on the isocitrate dehydrogenase (IDH) enzyme mutation status.

IDH is a ubiquitous enzyme that exists in three isoforms: IDH1 that is present in the cytosol, and IDH2 and IDH3 that are localized in the mitochondria [5]. IDH3 is involved in the normal Krebs cycle and has not been linked to tumorigenesis. Interestingly, the incidence of IDH1 and IDH2 mutations is elevated in gliomas. In fact, up to 80% of WHO grade II and III gliomas have IDH mutations, whereas only 5% of grade IV gliomas are IDH-mutant [6•]. Ninety-five percent of IDH mutations are found in the IDH1 isoform, with the most common mutation type consisting of a point mutation involving the arginine amino acid at codon 132, transforming it to histidine (R132H) in 92.7%, and less commonly to cysteine (R132C) in 3.6%, serine (R132S) in 1.8%, and glycine (R132G) in 0.9% [7]. The presence of IDH mutation in the vast majority of glioma cells and its higher prevalence in WHO grade II and III gliomas suggest that it is involved in the early steps of gliomagenesis [7]. In fact, three different pathways have been postulated for the development of gliomas. The first two pathways consist of an IDH mutation followed by either a mutation of the tumor suppressor gene TP53 and loss of transcriptional factor ATRX to differentiate into an astrocytoma, or loss of heterozygosity of chromosomes 1p and 19q (1p/19q codel) to form an oligodendroglioma. In the third pathway, the tumor cells retain the wild type form of IDH and rapidly acquire multiple complex genetic alterations with a tendency to progress into a glioblastoma (GBM), a WHO grade IV astrocytoma [5]. Further molecular studies identified mutations in TERT (telomerase reverse transcriptase), FUBP1 (far upstream element-binding protein 1), and CIC (capicua transcriptional repressor) in oligodendrogliomas [8, 9]. Tumor evolution studies have demonstrated that the development of an IDH mutation precedes the acquisition of other associated genetic events, such as TP53 mutation, suggesting it is an early, driver mutation [10–12].

An integrative genomic analysis, performed on 293 glioma patients by the Cancer Genome Atlas Network, showed that patients with oligodendrogliomas (IDH-mutant, 1p/19q codel) had a better overall survival (OS) than those with astrocytomas [13•, 14•], which was consistent with other reports [15]. However, IDH-mutant WHO grade II and III astrocytomas were associated with a better OS than their IDH wild-type counterparts, and, strikingly, IDH wild-type WHO grade II and III had a worse survival than IDH-mutant GBM. The survival advantage of IDH-mutant astrocytomas over their IDH wild-type counterparts has been also documented in several other reports [16••, 17], and WHO grade II and III astrocytic tumors with IDH mutation were found to have identical age at presentation and minimal difference in survival [17]. These findings led to a paradigm shift in the classification of glial tumors. Low-grade gliomas are not characterized by a lower histological grade anymore, but by their IDH-mutant status. Thus, oligodendrogliomas and IDH-mutant astrocytomas of WHO grade II and III can be grouped under the low-grade glioma category, whereas glioblastomas and IDH wild-type astrocytomas, which share similar molecular phenotypes, belong to the high-grade glioma group [13•].

## Epidemiology and Clinical Course

Low-grade gliomas account for 6.4% of all adult primary CNS tumors, with a predicted incidence in the USA of 0.51 and 0.25 per 100,000 per year for astrocytomas and oligodendrogliomas, respectively [18]. They occur more frequently in Whites than in Blacks, and even less frequently in American Indians/Alaska Natives and Asian/Pacific Islanders, and they are slightly more common in males. The peak incidence for astrocytomas is in the fourth decade, whereas oligodendrogliomas usually occur in the slightly older population [14•].

Besides a remote history of ionizing radiation [19], risk factors for the development of LGG, as is the case for high-grade gliomas, are poorly understood, although well-defined inherited tumor predisposition syndromes (e.g., neurofibromatosis type 1, Li-Fraumeni syndrome, Lynch syndrome) are believed to account for a small proportion of cases [20].

The majority of patients, particularly those with oligodendrogliomas, present with seizures, mainly of focal onset, while only about 30% present with focal neurological deficits such as aphasia or motor or sensory deficits. An even lower proportion presents with mental changes or signs of elevated intracranial pressure [21].

The diagnostic modality of choice for an initial evaluation of potential gliomas, like any other brain tumor, is a brain MRI. While many other entities present as T1 isointense or hypointense and T2 hyperintense on MRI, a systematic review and meta-analysis of several studies evaluating the imaging characteristics of more than 2000 pathologically proven LGG showed that, compared to IDH wild-type gliomas, IDH-mutant gliomas tend to be located in the frontal lobes, have well-defined borders, higher apparent diffusion coefficient, are minimally invasive on diffuse tensor imaging sequences, and have a low relative cerebral blood volume [22]. Another specific characteristic that was also described is the T2-FLAIR mismatch, where the tumor appears as diffusely hyperintense on T2 sequence, while it is mainly hypointense with a hyperintense rim on FLAIR sequence [23]. LGG usually do not enhance, and an area of enhancement usually indicates malignant progression, especially in astrocytomas [24]. However, 15 to 25% of LGG can show areas of patchy and faint or nodular-like enhancement [22]. Other imaging modalities were developed to increase the imaging diagnostic accuracy, the most relevant of which is magnetic resonance spectroscopy (MRS) that detects peaks of 2-hydroxyglutarate (2-HG), a product of a redox reaction catalyzed by a mutant IDH [25•]. This modality has been found to be more sensitive than others, with a sensitivity reaching up to 86%, while also being highly specific [26], but its sensitivity is highly dependent on the tumor volume and location.

The proliferation and progression of LGG are highly variable, and the median overall survival is around 10 to 11 years in astrocytomas and 15 years for oligodendrogliomas [13•, 14•]. While several factors have been proposed to affect the eventual prognosis, the following ones have been associated with an unfavorable outcome and a shorter survival: age at diagnosis of 40 years or older, astrocytoma histology subtype, presurgical largest tumor diameter of more than 6 cm, original tumor crossing the midline, a subtotal resection, and the presence of neurologic deficits before surgery [15]. Patients have been classically classified as low risk or high risk, depending on whether they have 2 or less, or more than 3 of these prognostic risk factors, respectively. Other poor prognostic factors have also been reported, including a rapid growth rate of more than 8 mm/year and a poor initial performance status [27]. More recent studies have reported that homozygous loss of chromosome 9p21 with loss of CDKN2A, a common phenomenon in higher WHO grade gliomas, is also associated with a worse outcome in IDH-mutant astrocytomas and oligodendrogliomas [28–30].

## Treatment

LGG remain a group of incurable diseases, and despite multiple efforts of studying and applying different therapeutic modalities, these tumors continue to result in premature death. However, some interventions have shown to improve median survival times.

Traditionally, LGG were considered a chronic and benign disease that affects young adults, rarely causing major neurological deficits besides seizures and having little impact on their quality of life. Hence, a “wait and see” approach was the standard of care. However, recent studies have shown that LGG grow continuously at a rate reaching 4 to 5 mm per year [31], and untreated symptomatic and incidentally discovered tumors will eventually undergo malignant transformation, leading to a more complicated disease course, a worse quality of life, and ultimately a shorter survival [32, 33]. Indeed, a Norwegian study followed 153 patients with LGG treated with either biopsy followed by watchful waiting or early surgical resection showed a 5-year OS of 60% and 74%, respectively, with the latter group surviving for 14.4 years on average, almost 2.5 times longer than the former [34, 35•]. Not only is biopsy alone associated with a shorter survival, it can lead to an underestimation of the tumor grade and misdiagnosis due to sampling error [2]. Furthermore, the extent of resection (EOR) was found to be positively associated with the outcome. A meta-analysis of 19 retrospective and one prospective studies addressing the relationship between EOR and prognosis in LGG showed a 4.6-fold increase in the median OS in patients who had any degree of surgical resection compared to those who only had a biopsy [36]. Another meta-analysis that included 3891 patients from 29 studies showed that surgical resection was associated with lower death and progression rates at different time points compared to biopsy, and those rates were lower in gross total resection (GTR) than in subtotal resection (STR) [37]. Resection of more than 80% of the tumor has been found to be acceptable for a better outcome in a study that included 216 patients; however, when safe, a complete resection of the FLAIR abnormality on MRI is the optimal surgical approach [38]. The introduction of intraoperative techniques like awake craniotomy, intraoperative MRI, the use of 5-aminolevulinic acid, and laser interstitial thermal therapy has made this goal more achievable [39].

Even after a gross total resection, however, the majority of patients, especially the ones classified as high risk, will have recurrence or progression of their tumor, hence the need for adjuvant therapy with radiation and chemotherapy. In the EORTC 22845 study, 290 patients were randomized to receive a total dose of 54 Gy of radiation either early after initial surgery or upon tumor progression. Postoperative imaging, let alone MRI, was not always available; EOR was assessed by the surgeon and differed among patients. Nonetheless, early radiation was associated with increased progression-free survival (PFS) but not OS [40]. Therefore, radiation can be spared until after disease progression in low-risk patients. However, early radiation should be considered in high-risk patients, and the usual dose used is 54 Gy given in 30 fractions of 1.8 Gy. This dose was based on an earlier study, EORTC 22844, along with other studies, showing no difference in OS and PFS in patients receiving moderate-dose (45 Gy) or high-dose of radiation (59.4 Gy) in the postoperative setting [37, 41].

Reports from the 1990s and early 2000s suggested that anaplastic oligodendrogliomas are chemosensitive, leading to an interest in treating patients with chemotherapy alone in order to potentially avoid long-term RT-induced toxicity. The EORTC 22033-26033 study, which compared the efficacy of RT alone to temozolomide alone (TMZ) in high-risk LGG showed no difference in progression-free survival between treatment arms [42]. However, at the same time, there was interest in exploring whether combination therapy was better than either single modality therapy. In recent years, increasing data have emerged from a long-term follow-up of a number of trials investigating this question, all supporting the superiority of combination therapy.

RTOG 9802 was among the first studies that assessed the efficacy of procarbazine/lomustine/vincristine (PCV) coupled to radiation in the treatment of WHO grade II high-risk LGG. It included 251 patients younger than 40 years of age with STR or patients above 40 years regardless of the EOR of their tumors. RT followed by PCV (RT + PCV) was associated with a better OS and PFS, and a lower risk of recurrence than RT alone, particularly in patients with oligodendrogliomas [43•]. Although the initial tumor classification relied on the histopathological diagnosis, retrospective molecular profiling revealed that the benefit of RT + PCV was only observed in IDH-mutant tumors, which constituted 60% of the total tumors. Additional results presented at the American Society for Radiation Oncology (ASTRO) meeting in 2019 also showed that median OS following combination therapy was longer in IDH-mutant, 1p/19q codel oligodendrogliomas [44].

Another study that was published around the same time, RTOG 9402, evaluated the effect of PCV followed by RT compared to RT alone in 291 patients with anaplastic oligodendrogliomas, as defined histologically. Although no difference in survival was observed between the two treatment arms in the entire population, a long-term follow-up revealed that combination therapy was associated with a better OS in patients with tumors with 1p/19q co-deletion [45•]. Concurrently, the EORTC 26951 study conducted in Europe compared RT followed by PCV to RT alone in a similar population of 368 anaplastic oligodendrogliomas. Interestingly, there was a significant prolongation of the median OS with RT + PCV compared to RT alone within the entire study population. Molecular profiling further revealed a trend towards benefit specifically for patients with tumors with 1p/19q co-deletion [46•].

Temozolomide has also been shown to have a benefit in the treatment of high-risk LGG. A phase II, single-arm study, evaluated the effect of TMZ used in the concurrent and adjuvant settings with RT. One hundred thirty-six patients received concurrent chemoradiation followed by up to 12 cycles of adjuvant TMZ. Overall survival at 3 years was higher than prespecified historical control values from EORTC 22844 [47]. The effect of TMZ was also assessed in a more recent larger study, the CATNON trial, where 745 patients with WHO Grade III IDH wild-type and IDH-mutant astrocytomas were randomized into receiving RT alone or followed by TMZ, or RT with concurrent TMZ with or without adjuvant TMZ [48•, 49]. Adjuvant TMZ increased median OS, and this effect was more evident in IDH-mutant tumors, whereas there was no benefit of concurrent TMZ in the entire cohort. However, there was a trend towards better outcomes from both concurrent and adjuvant TMZ in IDH-mutant astrocytomas.

Although both PCV and TMZ have shown efficacy in the treatment of LGG, both chemotherapies have been associated with grade 3 and 4 hematological toxicities. There are no mature data to date providing a head-to-head comparison of these two regimens in combination with radiation for the upfront treatment of high-risk LGG. A large prospective trial randomizing patients with high-risk LGG into receiving radiation alone vs chemotherapy alone, either TMZ or PCV, showed a survival advantage with chemotherapy in oligodendrogliomas but not in the entire cohort [50•]. Median PFS was similar in RT and PCV and was increased in these two groups compared to TMZ. There was no significant difference in OS between PCV and TMZ, although median PFS was longer in the oligodendroglioma group treated with PCV. Other retrospective studies have also compared PCV to TMZ with conflicting results [51–54]. ALLIANCE-N0577-CODEL is an ongoing trial comparing RT + TMZ to RT + PCV in anaplastic oligodendrogliomas with 1p/19q codeletion and may potentially provide a more definitive comparison between the two regimens.

## Novel perspectives in the treatment of low-grade gliomas

As mentioned earlier, the main characteristic of low-grade gliomas is their IDH mutation status. IDH1 and IDH2 are NADP+-dependent enzymes which catalyze the oxidative decarboxylation of isocitrate to α-ketoglutarate (α-KG) in the citric acid cycle [55]. However, a heterozygous mutation of IDH transforms α-KG to the R-isoform of 2-hydroxyglutarate (2-HG) through reductive carboxylation [56••]. IDH mutation is not specific to gliomas and has been found in other malignancies, namely acute myeloid leukemia, cholangiocarcinoma, chondrosarcoma, and myelodysplastic syndromes [57–61]. While it is associated with a worse outcome in some cancers like leukemias [62], it conveys a better prognosis in LGG, and IDH-mutant glioma cells exhibit a reduced proliferation rate compared to IDH-wildtype.

Unlike the S-isoform of 2-hydroxuglutarate that is physiologically induced by hypoxemia, the R-isoform is believed to be an oncometabolite [63]. In fact, 2-HG was found to alter several physiological cellular pathways. High levels of 2-HG inhibit histone demethylases and tet methylcytosine dioxygenase 2 enzyme (TET2), leading to histone and DNA hypermethylation, respectively [64–66]. 2-HG also inhibits the electron transport chain and interferes with amino acid metabolism by inhibiting branched-chain amino acid transferases [67, 68]. On the other hand, IDH mutation and elevated 2-HG levels inhibit certain anti-apoptotic proteins and DNA repair enzymes and reduce the intracellular glutathione level, leading to a lower threshold for apoptosis with enhanced sensitivity to alkylating agents and to radiation through reactive oxygen species [69–72].

Although radiation and alkylating chemotherapy have shown efficacy for the treatment of LGG, the response often lacks durability, and IDH-mutant tumors usually recur or progress to higher grade gliomas. Hence, there is much interest in finding other therapeutic modalities and novel therapies are emerging. Given that IDH mutation occurs early in the tumorigenesis process and is usually found in most tumor cells, targeting IDH1 mutation has been actively studied in the treatment of LGG. Inhibitors of the mutant IDH enzyme specifically target the production of 2-HG, but not of α-KG, and have been approved in the treatment of acute myeloid leukemia (AML) [73]. AG-120 (Ivosidenib) is currently under investigation for the treatment of recurrent or progressive IDH-mutant gliomas. Ivosidenib has been shown to have a strong selectivity to mutant IDH1 and led to reduced blood levels of 2-HG when it was used in a phase I study for treatment IDH-mutant solid tumors, including 66 recurrent or progressive gliomas [74]. Among these, 35 were non-enhancing gliomas, 19 of which were 1p/19q codeleted. Adverse events were frequent and included diarrhea, nausea, vomiting, headaches, anemia, neutropenia, and upper respiratory tract infections; 20% of patients experienced grade 3–4 adverse events [75•]. A minor response was observed in 2 patients, and 82.9% of patients had a stable disease, with more than half of the patients remaining on treatment for over 1 year. Another pan-mutant IDH1/2 inhibitor, AG-881 (Vorasidenib), was found to be highly potent and cross the blood-brain barrier. AG-881 was well tolerated in a phase I dose-escalation study (NCT02481154), where only 10 of 52 patients have grade 3 or 4 adverse events (seizures, transaminitis). Both Ivodisenib and Vorasidenib are currently being studied in a phase II trial for which preliminary results were presented at the Society of Neuro-Oncology (SNO) meeting in November 2019. Patients with IDH-mutant gliomas received drug 4 weeks before surgery and continued drug afterwards for a median of 6.93 months and 5.42 months, respectively. Forty-nine patients were randomized before surgery, and 39 remain on treatment. Objective tumor responses were observed in 31% of patients in each treatment group, and 2-HG levels in the resected tumor were 92% lower on average in patients who received neoadjuvant IDH inhibitors compared to those who did not. Only one patient experienced disease progression so far [76]. Based on these results, a phase III trial of vorasidenib has been launched in patients with grade II IDH-mutant gliomas progressing after surgery (INDIGO trial; NCT04164901). Another IDH1-mutant inhibitor, DS-1001b, previously studied in chondrosarcoma, is currently being evaluated in LGG in a phase I clinical trial (NCT03030066). Preliminary results presented at the 2019 American Society of Clinical Oncology (ASCO) meeting showed objective minor responses in 5 and partial responses in 1 out of 11 patients with non-enhancing tumors [77].

Other approaches of treating IDH-mutant gliomas do not involve direct inhibition of the IDH, but target pathways that are consequences of such mutation. As IDH mutation leads to reduced cellular capacity for double-stranded DNA breaks repair through interference with α-KG-dependent alkB homolog (ALKBH) and other DNA repair enzymes, the cells become reliant on alternative end-joining for DNA repair, a mechanism shared by BRCA1/BRCA2-deficient breast cancer cells [78]. This has raised the potential of a therapeutic benefit of poly(ADP-ribose) polymerase inhibitors (PARPi). Indeed, preclinical studies have shown that IDH mutant glioma cells are sensitive to PARPi [78] and that PARPi increase the sensitivity of GBM to TMZ [79–82]. Several ongoing phase I and II studies are using Olaparib alone or in combination with radiation and chemotherapy in the treatment of recurrent IDH-mutant and high-grade gliomas (NCT03561870, NCT03212742, NCT01390571). Another target that was considered in treatment of LGG is its hypermethylated status. CTCF insulator proteins normally function to preserve chromatin topography. It was shown that CTCF binding sites are methylated in IDH-mutant gliomas, leading to loss of CTCF binding and allowing interaction between platelet-derived growth factor A (PDGFRA) promoter and enhancer, leading to overexpression of PDGFRA [83]. This has been suggested as a potential mechanism by which IDH-mutant-induced hypermethylation leads to tumor progression. In preclinical models, demethylation of the CTCF domain led to the restoration of the insulation process, thereby preventing the upregulation of PDGFRA. Decitabine is a hypomethylating agent that is currently being studied in combination with cedazuridine, a cytosine deaminase inhibitor that prevents decitabine degradation in the gastrointestinal tract, in a phase I trial in recurrent/progressive non-enhancing IDH-mutant gliomas (NCT03922555).

Several other potential drugs are under investigation in the preclinical settings. Of interest is a glutaminase inhibitor, CB-839. 2-HG produced by mutant-IDH inhibits branched amino acid transferases, leading to depletion of cellular glutamate and, subsequently, the important antioxidant glutathione. IDH-mutant cells are therefore dependent on glutaminase to replete glutathione. In preclinical models, inhibition of glutaminase renders the IDH-mutant cells more sensitive to oxidative stress and, when combined with radiation, leads to cell death [68]. CB-839 is currently being tested in patients with IDH-mutant astrocytomas also receiving RT and temozolomide (NCT03528642).

In light of the striking efficacy in other tumor types, there has also been much interest in exploring immunotherapy approaches for lower grade gliomas. One strategy involves an IDH1 R132H-specific vaccine. In animal models, an IDH-mutant targeting vaccine generates an immune response and slows tumor growth [84, 85]. NOA-16 was a phase I clinical trial (NCT02454634) that evaluated safety and immunogenicity of an IDH1 R132H peptide vaccine administered to 32 patients with newly diagnosed grade III and grade IV IDH-mutant gliomas during the adjuvant temozolomide phase of treatment. One patient experienced a serious adverse advent that was attributed to the vaccine; the vaccine was otherwise well-tolerated in the remaining patients. Cellular and humoral immune responses were detected in 80% and 87% of patients, respectively. The investigators observed pseudoprogression in 12/32 (37.5%) of patients [86].

The immune checkpoint inhibitor avelumab, which targets the programmed death-ligand 1 (PD-L1) is being investigated in combination with hypofractionated radiation for patients with IDH-mutant gliomas that have transformed to grade IV following prior chemotherapy treatment (NCT02968940). A significant proportion of IDH-mutant gliomas that have previously been treated with alkylating chemotherapy develop a hypermutant phenotype [11, 12, 29], which could theoretically lead to increased neoantigen load in the tumor.

A major challenge to employing any immunotherapy approach in IDH-mutant gliomas is recent evidence that 2-HG produced by the mutant IDH enzyme creates an immunosuppressive tumor microenvironment [87, 88]. This raises the possibility that IDH inhibitors, which very effectively decrease 2-HG levels, could be given in combination with a vaccine or immune checkpoint inhibitor to enhance the immune response. Clinical trials testing this concept are still in the planning stages.

## Conclusion

Low-grade gliomas constitute a group of primary brain tumors that arise from glial cells. Their classification has evolved over time, and they are currently categorized by molecular rather than histopathological features. LGG consist of IDH-mutant WHO grade II and III gliomas, which can be further subcategorized into IDH-mutant, 1p/19 codeleted gliomas or oligodendrogliomas, and IDH-mutant, 1p/19q retained, p53-mutant, ATRX-mutant gliomas, or astrocytomas.

IDH-mutant gliomas usually affect adults in their fourth and fifth decades of life; although they have a better prognosis compared to their IDH wild-type counterpart gliomas and glioblastomas, they remain incurable and the vast majority recur, hence the need for aggressive treatment. A maximal safe surgical resection is the standard of care in the initial therapy, followed by 54 Gy of radiation and an alkylating agent-based chemotherapy in patients who are considered high risk, mainly being older than 40 years and/or having a subtotal resection. Both procarbazine/CCNU/vincristine (PCV) and temozolomide have shown survival benefit when added to radiation, but it remains unclear at this time whether these regimens are equivalent. A large prospective trial, the CODEL trial, is currently ongoing to address this issue. With the current standard of combination treatment, a median overall survival of 10 to 15 years has been achieved, with oligodendroglioma patients surviving longer than those with astrocytomas.

A number of novel, IDH-mutant-specific agents are currently under investigation or in the process of development, including IDH inhibitors, which have been approved for the treatment of acute myeloid leukemia, PARP inhibitors, and demethylating agents like decitabine. Other therapeutic modalities can be potentially used. Glutaminase inhibitors have shown to increase the IDH-mutant cell’s sensitivity to oxidative stress. Their combination to other therapies, namely chemotherapy and radiation, would be theoretically beneficial in the treatment of low-grade gliomas. Finally, given that CDKN2A mutations have been linked to a worse prognosis and a high likelihood of malignant progression, CDK4/6 inhibitors, which are currently used in breast cancer and have been studied in glioblastoma, can also be of interest in the treatment of patients with IDH-mutant gliomas. There is still a long way to go to find the best combination of therapies that prolongs the survival of those patients while having acceptable side effects and preserving a good quality of life.
